# Racial and Ethnic Differences in Receipt of Nonpharmacologic Care for Chronic Low Back Pain Among Medicare Beneficiaries With OUD

**DOI:** 10.1001/jamanetworkopen.2023.33251

**Published:** 2023-09-12

**Authors:** Fiona Bhondoekhan, Brandon D. L. Marshall, Theresa I. Shireman, Amal N. Trivedi, Jessica S. Merlin, Patience Moyo

**Affiliations:** 1 Department of Epidemiology, Brown University School of Public Health, Providence, Rhode Island; 2 Department of Health Services, Policy, and Practice, Brown University School of Public Health, Providence, Rhode Island; 3 CHAllenges in Managing and Preventing Pain Clinical Research Center, Division of General Internal Medicine, University of Pittsburgh, Pittsburgh, Pennsylvania

## Abstract

**Importance:**

Nonpharmacologic treatments are important for managing chronic pain among persons with opioid use disorder (OUD), for whom opioid and other pharmacologic therapies may be particularly harmful. Racial and ethnic minority individuals with chronic pain and OUD are vulnerable to suboptimal pain management due to systemic inequities and structural racism, highlighting the need to understand their receipt of guideline-recommended nonpharmacologic pain therapies, including physical therapy (PT) and chiropractic care.

**Objective:**

To assess differences across racial and ethnic groups in receipt of PT or chiropractic care for chronic low back pain (CLBP) among persons with comorbid OUD.

**Design, Setting, and Participants:**

This retrospective cohort study used a 20% random sample of national Medicare administrative data from January 1, 2016, to December 31, 2018, to identify fee-for-service community-dwelling beneficiaries with a new episode of CLBP and comorbid OUD. Data were analyzed from March 1, 2022, to July 30, 2023.

**Exposures:**

Race and ethnicity as a social construct, categorized as American Indian or Alaska Native, Asian or Pacific Islander, Black or African American, Hispanic, non-Hispanic White, and unknown or other.

**Main Outcomes and Measures:**

The main outcomes were receipt of PT or chiropractic care within 3 months of CLBP diagnosis. The time (in days) to receiving these treatments was also assessed.

**Results:**

Among 69 362 Medicare beneficiaries analyzed, the median age was 60.0 years (IQR, 51.5-68.7 years) and 42 042 (60.6%) were female. A total of 745 beneficiaries (1.1%) were American Indian or Alaska Native; 444 (0.6%), Asian or Pacific Islander; 9822 (14.2%), Black or African American; 4124 (5.9%), Hispanic; 53 377 (77.0%); non-Hispanic White; and 850 (1.2%), other or unknown race. Of all beneficiaries, 7104 (10.2%) received any PT or chiropractic care 3 months after a new CLBP episode. After adjustment, Black or African American (adjusted odds ratio, 0.46; 95% CI, 0.39-0.55) and Hispanic (adjusted odds ratio, 0.54; 95% CI, 0.43-0.67) persons had lower odds of receiving chiropractic care within 3 months of CLBP diagnosis compared with non-Hispanic White persons. Median time to chiropractic care was longest for American Indian or Alaska Native (median, 8.5 days [IQR, 0-44.0 days]) and Black or African American (median, 7.0 days [IQR, 0-42.0 days]) persons and shortest for Asian or Pacific Islander persons (median, 0 days [IQR, 0-6.0 days]). No significant racial and ethnic differences were observed for PT.

**Conclusions and Relevance:**

In this retrospective cohort study of Medicare beneficiaries with comorbid CLBP and OUD, receipt of PT and chiropractic care was low overall and lower across most racial and ethnic minority groups compared with non-Hispanic White persons. The findings underscore the need to address inequities in guideline-concordant pain management, particularly among Black or African American and Hispanic persons with OUD.

## Key Points

**Question:**

Are there racial and ethnic differences in receipt of physical therapy (PT) or chiropractic care for chronic low back pain (CLBP) among people with opioid use disorder (OUD)?

**Findings:**

In this cohort study of 69 362 Medicare beneficiaries with CLBP and OUD, 10.2% received PT or chiropractic services within 3 months. Black or African American and Hispanic persons had lower odds of chiropractic care compared with non-Hispanic White persons.

**Meaning:**

In this study, PT and chiropractic care use was low overall and racial and ethnic inequities in utilization and time to chiropractic care were observed, underscoring the need for equitable and multimodal pain management among people with OUD.

## Teaser

This cohort study assesses receipt of physical therapy and chiropractic care for chronic low back pain among Medicare beneficiaries with opioid use disorder (OUD) from different racial and ethnic groups.

## Introduction

Chronic pain and opioid use disorder (OUD) are closely linked: persons with chronic pain are at increased risk of OUD, and the prevalence of chronic pain among persons with OUD (64%) is higher than that in the general population (21%).^1,2,3^ It is critical to use chronic pain treatment strategies that are able to reduce not only the impact and duration of pain but also the mortality and morbidity from OUD.^4,5^ Several clinical practice guidelines have recommended a multimodal approach that prioritizes noninvasive, nonpharmacologic treatments for pain, such as physical therapy (PT) and chiropractic care.^6,7,8,9,10,11,12^

Managing chronic pain among persons with OUD, however, is complex as clinicians need to carefully consider guideline-recommended care for both conditions.^10,13^ Moreover, persons with chronic pain and OUD often have other undertreated comorbid substance use disorders, making them a population particularly susceptible to systemic and structural inequities.^14,15^ As such, negative perceptions of treating persons with overlapping chronic pain and OUD could potentially contribute to racial disparities in the delivery of guideline-concordant pain care.^16^ The racial and ethnic differences in pain care for persons with chronic pain and OUD are particularly important to investigate, as inequalities in health care access and quality of care can be compounded for racial and ethnic minority groups, contributing to lower receipt of guideline-concordant pain management than their non-Hispanic White counterparts. While the racial and ethnic gap in pain care has been well documented for pharmacologic therapies, few studies have examined whether racial and ethnic disparities exist in receipt of PT and chiropractic care for chronic pain,^17,18,19,20,21^ and, to our knowledge, this question has not been assessed among persons with OUD.

The objective of this study was to investigate differences in use of PT and chiropractic care among persons diagnosed with chronic pain and OUD across racial and ethnic groups. We analyzed a cohort of Medicare beneficiaries, a population with high reported prevalence of chronic pain among whom rates of OUD are increasing^22,23,24^ and who had access to Medicare-covered nonpharmacologic treatments, such as PT and chiropractic care. We hypothesized that following a new diagnosis of chronic pain, racial and ethnic minority groups would have lower receipt of PT and chiropractic care compared with non-Hispanic White individuals and that among persons who receive these treatments, racial and ethnic minority groups would have a longer time to receipt of treatment.

## Methods

The Brown University institutional review board determined the study to be exempt from review based on secondary analysis of deidentified data, and informed consent was not required. This study followed the Strengthening the Reporting of Observational Studies in Epidemiology (STROBE) reporting guideline.

### Study Data and Population

We conducted a retrospective cohort study using a 20% random sample of national Medicare administrative data from January 1, 2016, to December 31, 2018, linked to publicly available data on community and practitioner characteristics. Diagnoses and dates of service utilization came from MedPAR Part A and Part B claims, excluding long-term and post–acute care settings. Prescribed medications were obtained from Medicare Part D claims, and the Medicare enrollment file contained information on demographics, enrollment year, and geographic location.

The study population included Medicare fee-for-service beneficiaries with 2 or more diagnoses for chronic low back pain (CLBP) separated by at least 90 days^25^ from July 1, 2016, to June 30, 2018, with the first claim assigned as the index date.^26^ We focused on CLBP as it is one of the most prevalent pain conditions in the US.^27^ We identified CLBP using *International Statistical Classification of Diseases and Related Health Problems, Tenth Revision (ICD-10)* codes for radicular and nonradicular back pain in any position on MedPAR Part A and Part B claims (eTable 1 in Supplement 1).^28^ A 6-month look-back period was applied to ensure no CLBP claims occurred prior to the index date; as such, the diagnosis on the index date was considered a new episode of CLBP. When beneficiaries had multiple eligible CLBP episodes during the study period, we randomly selected 1 episode for inclusion in the analysis. We assumed OUD to be present if participants had 1 or more MedPAR Part A or Part B claim for “opioid abuse” or “opioid dependence” at any time from January 1, 2016, to December 31, 2018, to account for potential underdiagnosis of OUD within claims data. Beneficiaries with any of the following criteria were excluded: not continuously enrolled in Medicare for 12 months; enrolled in Medicare Advantage (due to incomplete claims); in hospice or long-term care facility; 2 or more claims for a cancer diagnosis (treatment for cancer-related chronic pain is distinct from that for noncancer chronic pain and was not the focus of this study)^25,29^; younger than 18 years; no OUD claims during study period; residing outside the 50 states and Washington, DC, including Puerto Rico, US Virgin Islands, American Samoa, Marianas Islands, and Guam; or missing Federal Information Processing System Codes for States and Counties.

### Outcome

The main outcomes of interest were 2 binary variables representing receipt of active PT or chiropractic care 3 months after the CLBP diagnosis. A third outcome representing receipt of either of these services was also created. Physical therapy and chiropractic care were 2 evidence-based nonpharmacologic pain therapies covered by Medicare during the study period and were identified using *Current Procedural Terminology* (*CPT*) codes from Part B claims (eTable 2 in Supplement 1). For PT, only *CPT* codes identifying treatment modalities and therapeutic procedures were included, while *CPT* codes for evaluations were excluded from the main analysis as they were deemed not to represent receipt of an active intervention. Claims with both evaluations and treatment modalities or therapeutic procedures billed on the same day were considered to indicate receipt of active PT. The *CPT* codes for PT and/or chiropractic care occurring on the day of CLBP diagnosis were included, as beneficiaries often receive therapeutic or chiropractic services at the time of CLBP diagnosis in outpatient settings. The time to receipt of PT and chiropractic care within the 3-month follow-up period was calculated in days from CLBP diagnosis.

### Primary Independent Variable

The primary independent variable was beneficiary race and ethnicity, which we considered as a social rather than a biologic or genetic construct.^30^ We used the Research Triangle Institute (RTI) race and ethnicity variable in Medicare enrollment files, which relies on administrative data from the Social Security Administration and an algorithm to impute race and ethnicity from name and geographic location. The validity of the race and ethnicity variables in Medicare data compared with the gold standard of self-report has been evaluated, and findings indicate that the RTI race code is more accurate for Asian or Pacific Islander and Hispanic persons than the Medicare enrollment file beneficiary race code.^31,32^ The RTI variable reports race and ethnicity in 7 mutually exclusive groups as American Indian or Alaska Native, Asian or Pacific Islander, Black or African American, Hispanic, non-Hispanic White, other, and unknown. The other category refers to non-Hispanic other race, and a designation of unknown occurs when there are missing data.^33^ Due to small numbers in the unknown group, we combined it with the group labeled as other, therefore creating a 6-level categorical variable identifying beneficiary race and ethnicity.

### Covariates

Beneficiary characteristics, such as age, sex (as recorded in the enrollment file), current reason for Medicare eligibility, dual eligibility, state and county of residence, and receipt of medication for OUD were measured in the year of CLBP diagnosis from the annual Medicare enrollment file. Comorbidities included cardiovascular conditions, mental health diagnosis, neck pain and fractures, osteoarthritis and joint cartilage conditions, diabetes, chronic obstructive pulmonary disease, obesity, alcohol use disorder, hospitalization, surgery, and the Gagne comorbidity score. All were measured 6 months prior to the CLBP diagnosis date using *ICD-10* or *CPT* codes from MedPAR Part A and Part B claims (eTable 3 in Supplement 1).^28,34,35,36,37^ Opioid prescriptions, excluding medications for OUD, in the 6 months prior to the CLBP diagnosis date were obtained from Part D claims (eTable 4 in Supplement 1).

We used the American Community Survey^38^ to create variables for social determinants of health at the county level and the Area Health Resources File^39^ to determine geographic information and practitioner availability at the state level (eTable 5 in Supplement 1). Social determinants of health were based on 7 demographic characteristics in the Social Deprivation Index to identify areas at the county level in need for health care resources.^40^ These variables were calculated using 5-year county-level American Community Survey estimates from 2014 to 2018.^40^ State-level practitioner availability included the number of physical therapists (including assistants and aides) and number of chiropractors per 100 000 population in the year of CLBP diagnosis and came from the Area Health Resources File. We linked National Plan and Provider Enumeration System^41^ data to the billing health care practitioner in the Part B claims using a National Provider Identifier to identify practitioner specialty. Lastly, US region and rural-urban commuting area codes were obtained from the Area Health Resources File at the county level.^39^ No missing data were observed for individual-level (demographic, comorbidities, and medication), county-level (social determinants of health), or state-level (practitioner availability, practitioner specialty) data.

### Statistical Analysis

Characteristics of the study population were compared by race and ethnicity using the Kruskal-Wallis test for continuous variables and Pearson χ^2^ test for binary and categorical variables. We calculated the proportions of beneficiaries within each race and ethnicity group who received (1) any PT or chiropractic care, (2) any PT, and (3) any chiropractic care within 3 months of CLBP diagnosis and used Kaplan-Meier curves to visualize the time to receipt of these treatments. Differences across race and ethnicity in the Kaplan-Meier curves were evaluated using log-rank tests. Multilevel logistic regression models separately estimated the association between race and ethnicity and the 3 outcomes, adjusting for all covariates listed in Table 1 and eTable 6 in Supplement 1 in addition to receipt of any PT or chiropractic care prior to CLBP diagnosis and addiction specialist encounters during follow-up. State- and county-level variation were accounted for by inclusion of individual random intercepts for states and counties in each model. The results of the models are presented as adjusted odds ratios (AORs) with accompanying 95% CIs.

**Table 1.** Characteristics of the Study Population by Race and Ethnicity

Characteristic	Beneficiaries, No. (%)					
	American Indian or Alaska Native (n = 745)	Asian or Pacific Islander (n = 444)	Black or African American (n = 9822)	Hispanic (n = 4124)	Non-Hispanic White (n = 53 377)	Unknown or other (n = 850)^a^
Age						
Median (IQR)	57.4 (48.9-66.3)	65.2 (51.6-73.7)	57.5 (49.7-65.5)	57.4 (48.2-66.9)	60.8 (52.2-69.3)	62.7 (46.8-68.4)
≥65 y	212 (28.5)	223 (50.2)	2588 (26.3)	1276 (30.9)	21 499 (40.3)	391 (46.0)
Sex						
Female	496 (66.6)	266 (59.9)	6220 (63.3)	2501 (60.6)	32 147 (60.2)	412 (48.5)
Male	249 (33.4)	178 (40.1)	3602 (36.7)	1623 (39.4)	21 230 (39.8)	438 (51.5)
Comorbidities^b^						
Cardiovascular condition^c^	440 (59.1)	304 (68.5)	7578 (77.2)	2629 (63.7)	33 125 (62.1)	487 (57.3)
Mental health diagnosis^d^	356 (47.8)	189 (42.6)	4399 (44.8)	2205 (53.5)	27 756 (52.0)	411 (48.4)
Neck pain	255 (34.2)	152 (34.2)	3131 (31.9)	1434 (34.8)	18 492 (34.6)	280 (32.9)
Fractures	55 (7.4)	30 (6.8)	329 (3.4)	206 (5.0)	3184 (6.0)	54 (6.4)
Osteoarthritis and joint cartilage conditions	290 (38.9)	162 (36.5)	4255 (43.3)	1458 (35.4)	18 782 (35.2)	298 (35.1)
Diabetes	253 (34.0)	173 (39.0)	3911 (39.8)	1623 (39.4)	13 750 (25.8)	232 (27.3)
Chronic obstructive pulmonary disease	175 (23.5)	85 (19.1)	2172 (22.1)	739 (17.9)	13 509 (25.3)	147 (17.3)
Obesity	150 (20.1)	60 (13.5)	2631 (26.8)	946 (22.9)	9050 (17.0)	148 (17.4)
Alcohol use disorder	26 (3.5)	13 (2.9)	440 (4.5)	143 (3.5)	2099 (3.9)	51 (6.0)
Hospitalization	133 (17.9)	67 (15.1)	1888 (19.2)	744 (18.0)	8723 (16.3)	157 (18.5)
Surgery^e^	35 (4.7)	21 (4.7)	445 (4.5)	170 (4.1)	2756 (5.2)	37 (4.4)
Gagne comorbidity score						
≤0	304 (40.8)	167 (37.6)	3814 (38.8)	1532 (37.2)	21 614 (40.5)	357 (42.0)
1	175 (23.5)	83 (18.7)	1995 (20.3)	931 (22.6)	12 324 (23.1)	182 (21.4)
2-3	165 (22.2)	113 (25.5)	2215 (22.6)	954 (23.1)	12 066 (22.6)	180 (21.2)
≥4	101 (13.6)	81 (18.2)	1798 (18.3)	707 (17.1)	7373 (13.8)	131 (15.4)
Medication						
Opioid prescription^b^	685 (92.0)	365 (82.2)	8915 (90.8)	3592 (87.1)	46 805 (87.7)	686 (80.7)
MOUD^f^	37 (5.0)	18 (4.1)	291 (3.0)	195 (4.7)	3250 (6.1)	54 (6.4)
Geographic location^f^						
Urbanicity						
Metropolitan	403 (54.1)	410 (92.3)	8324 (84.7)	3628 (88.0)	38 519 (72.2)	679 (79.9)
Urban	303 (40.7)	32 (7.2)	1288 (13.1)	470 (11.4)	12 986 (24.3)	158 (18.6)
Rural	39 (5.2)	NR	210 (2.1)	26 (0.6)	1872 (3.5)	13 (1.5)
US region						
Midwest	111 (14.9)	44 (9.9)	2049 (20.9)	346 (8.4)	10 068 (18.9)	168 (19.8)
Northeast	17 (2.3)	66 (14.9)	1145 (11.7)	817 (19.8)	8139 (15.2)	218 (25.6)
South	401 (53.8)	131 (29.5)	5662 (57.6)	1428 (34.6)	26 269 (49.2)	277 (32.6)
West	216 (29.0)	203 (45.7)	966 (9.8)	1533 (37.2)	8901 (16.7)	187 (22.0)

Abbreviations: MOUD, medication for opioid use disorder; NR, not reported (sample size <11).

^a^ Other refers to non-Hispanic other races, and any missing values were coded as unknown.

^b^ Assessed 6 months prior to the date of chronic low back pain diagnosis.

^c^ Defined as a diagnosis of heart failure, ischemic heart disease, or hypertension.

^d^ Defined as a diagnosis of depressive disorder, anxiety disorder, or schizophrenia or related disorder.

^e^ Refers to 24 procedures associated with postsurgical pain.

^f^ Assessed in the year of chronic low back pain diagnosis.

We conducted 2 sensitivity analyses. First, we modified the PT outcome to include *CPT* codes for PT evaluations in addition to treatment modalities and therapeutic procedures because of the potential for beneficiaries to obtain clinical benefit through their interaction with a practitioner during the evaluation. Second, to examine the influence of prior PT or chiropractic care on cohort selection, we restricted the study cohort to beneficiaries without any PT or chiropractic care in the 6 months before CLBP diagnosis. Persons with prior use might have different experiences and attitudes toward nonpharmacologic care that increase their continued engagement or disengagement in care; these experiences could also differ by race and ethnicity.

All analyses were conducted in SAS, version 9.4 (SAS Institute Inc) and Stata, version 17.0 (StataCorp LLC), and statistical significance was evaluated at *P* < .05 (2-sided test). Data were analyzed from March 1, 2022, to July 30, 2023.

## Results

### Study Population Description

**Figure 1.**
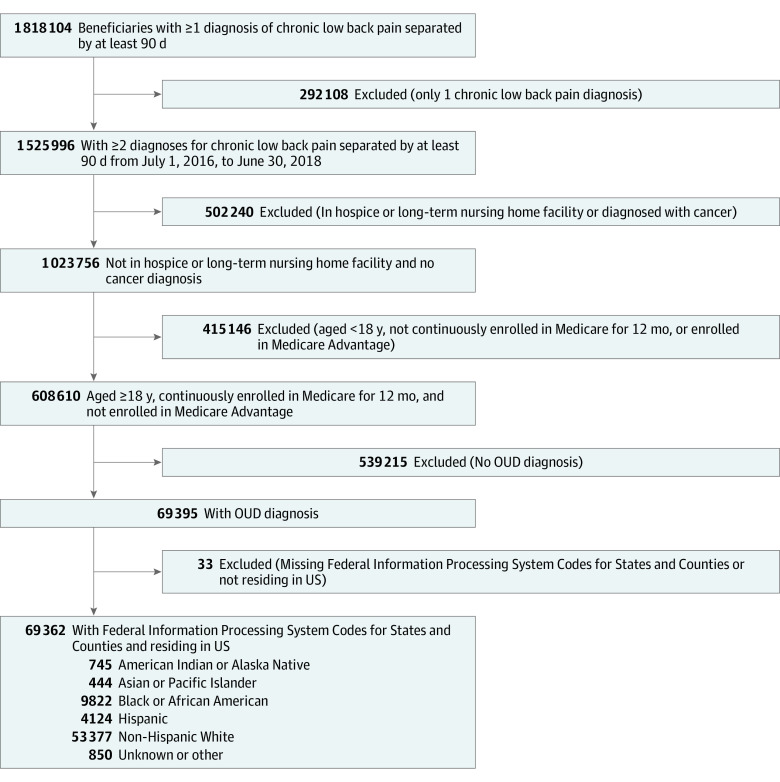
Participant Flowchart OUD indicates opioid use disorder.

Among the 69 362 beneficiaries diagnosed with CLBP and OUD (Figure 1), the median age was 60.0 years (IQR, 51.5-68.7 years), 42 042 (60.6%) were female, and 27 320 (39.4%) were male (eTable 7 in Supplement 1). In terms of race and ethnicity, 745 (1.1%) were American Indian or Alaska Native; 444 (0.6%), Asian or Pacific Islander; 9822 (14.2%), Black or African American; 4124 (5.9%), Hispanic, and 53 377 (77.0%) non-Hispanic White. For 850 beneficiaries (1.2%), no race and ethnicity was reported in the enrollment file (unknown or other). The characteristics of the study population by race and ethnicity are outlined in Table 1. Cardiovascular conditions and mental health diagnoses were common among beneficiaries from all race and ethnicity groups. While opioid prescription for pain was common for all groups, only 3845 of 69 362 beneficiaries (5.5%) used medication for OUD, with lowest use in Black or African American persons (291 of 9822 [3.0%]). The distribution of county-level social determinants of health, state-level practitioner availability, and specialists engaged during follow-up did not differ markedly by race and ethnicity (eTables 6 and 8 in Supplement 1).

### Nonpharmacologic Treatment Use

Three months after CLBP diagnosis, the prevalence of PT was low but higher (4358 [6.3%]) compared with chiropractic care (3227 [4.7%]). Use of PT was lowest among American Indian or Alaska Native persons (33 of 745 [4.4%]) and highest among Asian or Pacific Islander persons (47 of 444 [10.6%]) (Table 2). Chiropractic care was lowest among Black or African American (170 of 9822 [1.7%]) and Hispanic (98 of 4124 [2.4%]) persons and highest among non-Hispanic White persons (2865 of 53 377 [5.4%]) and those in the unknown or other group (51 of 850 [6.0%]).

**Table 2.** Treatment Use by Race and Ethnicity

Treatment	Beneficiaries					
	American Indian or Alaska Native (n = 745)	Asian or Pacific Islander (n = 444)	Black or African American (n = 9822)	Hispanic (n = 4124)	Non-Hispanic White (n = 53 377)	Unknown or other (n = 850)^a^
Any physical therapy or chiropractic care						
Use prior to CLBP diagnosis, No. (%)^b^	105 (14.1)	100 (22.5)	1411 (14.4)	648 (15.7)	9447 (17.7)	176 (20.7)
Use 3 mo after CLBP diagnosis, No. (%)^b^	57 (7.7)	61 (13.7)	713 (7.3)	370 (9.0)	5783 (10.8)	120 (14.1)
Time to treatment, median (IQR), d^b^	9.0 (0-40.0)	6.0 (0-30.0)	13.0 (0-43.0)	8.5 (0-39.0)	5.0 (0-34.0)	6.0 (0-30.5)
Any physical therapy						
Use 3 mo after CLBP diagnosis, No. (%)^b^	33 (4.4)	47 (10.6)	582 (5.9)	283 (6.9)	3335 (6.2)	78 (9.2)
Time to treatment, median (IQR), d	14.0 (1.0-39.0)	14.0 (1.0-49.0)	16.0 (2.0-47.0)	14.0 (1.0-43.0)	15.0 (1.0-48.0)	18.5 (1.0-42.0)
Any chiropractic care						
Use 3 mo after CLBP diagnosis, No. (%)^b^	26 (3.5)	17 (3.8)	170 (1.7)	98 (2.4)	2865 (5.4)	51 (6.0)
Time to treatment, median (IQR), d^b^	8.5 (0-44.0)	0 (0-6.0)	7.0 (0-42.0)	0 (0-33.0)	0 (0-21.0)	0 (0-16.0)

Abbreviation: CLBP, chronic low back pain.

^a^ Other refers to non-Hispanic other races, and any missing values were coded as unknown.

^b^ *P* < .05.

**Figure 2.**
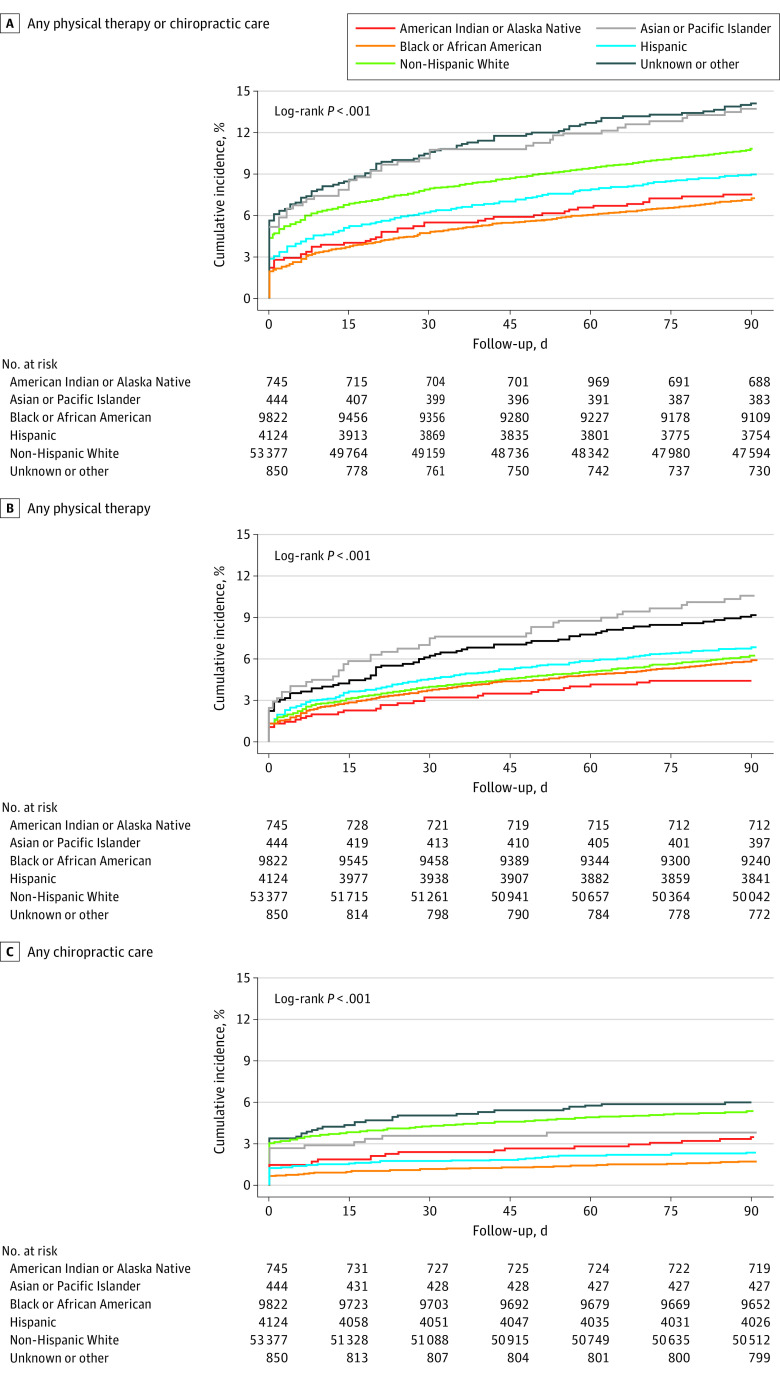
Cumulative Incidence of Physical Therapy and Chiropractic Care by Race and Ethnicity

Median time to PT initiation did not differ substantially across racial and ethnic groups; however, American Indian or Alaska Native persons (median, 8.5 days [IQR, 0-44.0 days]) and Black or African American persons (median, 7.0 days [IQR, 0-42.0 days]) had a longer time to receipt of chiropractic care compared with non-Hispanic White persons (median, 0 days [IQR, 0-21.0 days]). Cumulative incidence curves (Figure 2) indicate that the differences in chiropractic care across race and ethnicity occurred in the early period following CLBP diagnosis and widened over time.

### Differences in Nonpharmacologic Treatment

The multilevel logistic regression models showed lower odds of any PT or chiropractic care among American Indian or Alaskan Native (AOR, 0.74; 95% CI, 0.54-0.99), Black or African American (AOR, 0.79; 95% CI, 0.71-0.87), and Hispanic (AOR, 0.84; 95% CI, 0.74-0.96) persons compared with non-Hispanic White persons after adjustment (Table 3 and eTable 9 in Supplement 1). Separate analysis of any PT and any chiropractic care as outcomes revealed that these findings were driven by lower odds of any chiropractic care use among Black or African American (AOR, 0.46; 95% CI, 0.39-0.55) and Hispanic (AOR, 0.54; 95% CI, 0.43-0.67) persons (eTable 10 in Supplement 1). Receipt of any PT did not differ across racial and ethnic groups after adjustment (eTable 11 in Supplement 1).

**Table 3.** Multilevel Logistic Regression for the Association Between Physical Therapy and Chiropractic Care With Race and Ethnicity

Race and ethnicity	Odds ratio (95% CI)					
	Any physical therapy or chiropractic care		Any physical therapy		Any chiropractic care	
	Unadjusted	Adjusted^a^	Unadjusted	Adjusted^a^	Unadjusted	Adjusted^a^
American Indian or Alaska Native	0.64 (0.48-0.85)^b^	0.74 (0.54-0.99)^c^	0.73 (0.51-1.05)	0.81 (0.55-1.17)	0.54 (0.36-0.81)^b^	0.69 (0.45-1.06)
Asian or Pacific Islander	1.06 (0.80-1.41)	0.96 (0.70-1.31)	1.26 (0.92-1.73)	1.07 (0.76-1.51)	0.71 (0.40-1.16)	0.77 (0.45-1.29)
Black or African American	0.66 (0.61-0.72)^d^	0.79 (0.71-0.87)^d^	0.89 (0.80-0.98)^c^	1.02 (0.92-1.14)	0.36 (0.30-0.42)^d^	0.46 (0.39-0.55)^d^
Hispanic	0.69 (0.61-0.77)^d^	0.84 (0.74-0.96)^b^	0.85 (0.74-0.97)^c^	1.00 (0.87-1.15)	0.42 (0.34-0.52)^d^	0.54 (0.43-0.67)^d^
Non-Hispanic White	1 [Reference]	1 [Reference]	1 [Reference]	1 [Reference]	1 [Reference]	1 [Reference]
Unknown or other^e^	1.17 (0.96-1.43)	1.08 (0.86-1.36)	1.24 (0.97-1.58)	1.20 (0.92-1.55)	1.05 (0.79-1.41)	0.90 (0.65-1.23)

^a^ Adjusted models included demographic characteristics, reason for Medicare eligibility, dual eligibility, any physical therapy or chiropractic care prior to chronic low back pain diagnosis, comorbidities, medication, social determinants of health, state-level practitioner availability, urbanicity, US region, and addiction specialist encounters during follow-up. All models accounted for clustering by county and state.

^b^ *P* < .01.

^c^ *P* < .05.

^d^ *P* < .001.

^e^ Other refers to non-Hispanic other races, and any missing values were coded as unknown.

### Sensitivity Analyses

The first sensitivity analysis including a broader definition of PT yielded similar results as the main analysis (eTable 12 in Supplement 1). The same models were repeated to obtain estimates for the association between race and ethnicity and any PT, and the associations remained unchanged. The results of a second sensitivity analysis that excluded beneficiaries who received any PT or chiropractic care in the 6 months prior to CLBP diagnosis (n = 11 887) were also similar to the results for receipt of any PT or chiropractic care; however, only the association for Black or African American persons remained (AOR, 0.84; 95% CI, 0.73-0.96) (eTable 13 in Supplement 1).

## Discussion

In this retrospective cohort study of Medicare beneficiaries with a new episode of CLBP and comorbid OUD, we found that receipt of PT and chiropractic care was minimal, with approximately 1 in 10 beneficiaries receiving either of these services within 3 months of CLBP diagnosis. Our findings point to differential receipt of chiropractic care within subgroups of race and ethnicity. Specifically, Black or African American and Hispanic persons had significantly lower odds of chiropractic care compared with non-Hispanic White persons. These results add to the small but emerging body of literature on use of nonpharmacologic pain treatment in the US among persons with comorbid CLBP and OUD.

There is little literature in general regarding nonpharmacologic treatment use and disparities, and the few existing studies indicate wide variations across racial and ethnic subgroups.^42,43,44,45,46^ Our study found that pervasive racial and ethnic differences in chronic pain treatment extended to chiropractic care and persons with OUD. We also shed light on the utilization patterns of PT and chiropractic care by evaluating the time to treatment, which revealed disparities in access for American Indian or Alaska Native and Black or African American persons that increased over time and were primarily observed for chiropractic care. Interestingly, the cumulative incidence and time to PT in our study was consistent with other reports from large national databases.^47,48^ Fritz et al^47^ reported a PT prevalence of 7% within 90 days of the back pain visit for patients with commercial health insurance, with median time to use of PT of 14 days (IQR, 6-33 days). Compared with other studies evaluating musculoskeletal chronic pain, however, we observed lower use of chiropractic care.^27,45,49,50^ The geographic availability of practitioners may contribute to these differential patterns of PT and chiropractic care.^51^ It has been reported that labor supply of both PT and chiropractic care is lower in the South and West regions of the US compared with the Northeast and Midwest.^52,53^ Variations in local practitioner availability possibly limit access, acceptability, and adoption of these types of pain treatments.^42,53^ Limited patient and practitioner knowledge combined with skepticism about the efficacy of nonpharmacologic pain treatment and cultural influences on patient preferences may also impede uptake of these therapies, particularly among patients already receiving opioid therapy.^17,54^

Our findings also call attention to barriers in accessing and using PT and chiropractic care for chronic pain that are compounded by the intersectional nature of racism and OUD-related stigma. Harmful biases about racial and ethnic minority groups with OUD are reinforced in health care settings, resulting in these groups receiving fewer referrals to pain specialists and consequently fewer opportunities to receive nonpharmacologic treatments compared with non-Hispanic White individuals.^55,56^ The new clinical practice guidelines for prescribing opioids for pain by the Centers for Disease Control and Prevention recommend that clinicians provide evidence-based medications for persons with OUD.^10^ Incorporating nonpharmacologic pain therapies along with medication for OUD creates a pain management approach that can minimize safety risks. Accordingly, several trials using behavioral and mind-based therapies to reduce pain and opioid use are currently under way.^57,58^ Efforts should also prioritize a multidisciplinary and patient-centered approach that involves cultural competency and understanding of systemic and structural inequities to ensure equal opportunities in pain and OUD care.^59^ Current health care policies in coverage and financing in Medicare systematically prevent racial and ethnic minority groups from accessing high-quality care.^60^ Evidence has consistently demonstrated that Black or African American and Hispanic persons are less likely to receive evidence-based care and age-appropriate diagnostic screenings.^61^ In addition, practitioners that primarily care for low-income minority groups are more likely to receive lower reimbursements from Medicare under value-based payment programs, while pay-for-performance programs reward practitioners caring for high-income and non-Hispanic White individuals.^62^ To this end, clinical trials assessing nonpharmacologic treatments of chronic pain and OUD can enlist community partnerships to assist in creating effective, safe, and equitable strategies for treatment of both conditions.^63^ Additionally, current guidelines can prioritize addressing racial and ethnic inequities in treatment of chronic pain and OUD through comprehensive pain clinical care that incorporates factors at the individual, practitioner, and system levels that contribute to the observed disparities.

### Limitations

Several limitations of the study should be considered. First, Medicare claims data are limited in their ability to accurately determine race and ethnicity. However, in the absence of self-report, the RTI algorithm used is the best alternative.^64^ Exclusion of beneficiaries residing in Puerto Rico and the US Virgin Islands also limited the study sample of Hispanic persons. However, as most beneficiaries from these territories are enrolled in Medicare Advantage, they would not have met our inclusion criteria. Second, lack of information on whether practitioners differentially referred beneficiaries to PT or chiropractic care (along with individual perspectives and preferences) limits understanding of the underlying mechanisms that explain differences in use of these treatments. Third, due to limited Medicare coverage of nonpharmacologic pain therapies, we could not assess a broader list of treatments aside from PT and chiropractic care. We attempted to characterize cognitive behavioral therapies, but low prevalence limited further assessment by race and ethnicity. Fourth, differential OUD diagnosis by practitioners based on beneficiaries’ racial demographics may contribute to under- or overestimation of the differences in PT or chiropractic care for Black or African American and Hispanic persons, as these groups historically have been underdiagnosed for OUD.^65^ Fifth, a limitation inherent with administrative claims data is that we could not capture self-paid services for which Medicare did not provide reimbursement, leading to possible underestimation of chronic pain services among beneficiaries.

## Conclusions

This cohort study found that receipt of PT and chiropractic care was low overall and lower across most racial and ethnic minority groups compared with non-Hispanic White individuals despite nonpharmacologic approaches to pain management being widely recommended as a strategy to mitigate opioid-related morbidity and mortality. Although there is uncertainty about the individual and practitioner contexts that may explain our findings, historic racial and ethnic disparities in pain treatment appear to persist and are also specifically evident for chiropractic care in persons with OUD. Therefore, efforts are needed to elucidate and address the individual, practitioner, and system-level risk factors that limit racial equity in guideline-recommended pain management in general and for persons with OUD.

## Notes

Supplement 1. **eTable 1.** *ICD-10* codes for chronic back pain and opioid use disorder from MedPAR Part A and Part B claims **eTable 2.** Physical therapy and chiropractic care *CPT* codes from Part B claims **eTable 3.** *ICD-10* and *CPT* codes for comorbidities from MedPAR Part A and Part B claims **eTable 4.** Generic drug names for opioid prescriptions, excluding buprenorphine, in Part D claims **eTable 5.** Data sources and definitions for county-level social determinants of health, geographic characteristics, and state-level practitioner availability **eTable 6.** Additional characteristics of the study population by race and ethnicity **eTable 7.** Characteristics of overall study population **eTable 8.** Practitioner encounters after chronic low back pain diagnosis by race and ethnicity **eTable 9.** All multilevel logistic regressions for the association between any physical therapy or chiropractic care and race and ethnicity **eTable 10.** All multilevel logistic regressions for the association between any chiropractic care and race and ethnicity **eTable 11.** All multilevel logistic regressions for the association between any physical therapy and race and ethnicity **eTable 12.** All multilevel logistic regressions for the association between any physical therapy (including n = 209 with evaluations only) and race and ethnicity **eTable 13.** All multilevel logistic regressions for the association between any physical therapy or chiropractic care and race and ethnicity (excluding 11 887 individuals with any physical therapy or chiropractic care prior to chronic low back pain diagnosis)

Supplement 2.
Data Sharing Statement
