# Supplementary material for: Racial and Ethnic Differences in Receipt of Nonpharmacologic Care for Chronic Low Back Pain Among Medicare Beneficiaries With OUD
Source: JAMA Netw Open. 2023 Sep 12;6(9):e2333251. doi: 10.1001/jamanetworkopen.2023.33251 (PMC10498328; doi:10.1001/jamanetworkopen.2023.33251)
Supplement: Supplement 1. — eTable 1. ICD-10 codes for chronic back pain and opioid use disorder from MedPAR Part A and Part B claims eTable 2. Physical therapy and chiropractic care CPT codes from Part B claims eTable 3. ICD-10 and CPT codes for comorbidities from MedPAR Part A and Part B claims eTable 4. Generic drug names for opioid prescriptions, excluding buprenorphine, in Part D claims eTable 5. Data sources and definitions for county-level social determinants of health, geographic characteristics, and state-level practitioner availability eTable 6. Additional characteristics of the study population by race and ethnicity eTable 7. Characteristics of overall study population eTable 8. Practitioner encounters after chronic low back pain diagnosis by race and ethnicity eTable 9. All multilevel logistic regressions for the association between any physical therapy or chiropractic care and race and ethnicity eTable 10. All multilevel logistic regressions for the association between any chiropractic care and race and ethnicity eTable 11. All multilevel logistic regressions for the association between any physical therapy and race and ethnicity eTable 12. All multilevel logistic regressions for the association between any physical therapy (including n = 209 with evaluations only) and race and ethnicity eTable 13. All multilevel logistic regressions for the association between any physical therapy or chiropractic care and race and ethnicity (excluding 11 887 individuals with any physical therapy or chiropractic care prior to chronic low back pain diagnosis) [file jamanetwopen-e2333251-s001.pdf]

Supplemental Online Content

Bhondoekhan F, Marshall BDL, Shireman TI, Trivedi AN, Merlin JS, Moyo P. Racial and ethnic differences in receipt of nonpharmacologic care for chronic low back pain among Medicare beneficiaries with OUD. *JAMA Netw Open.* 2023;6(9):e2333251.
doi:10.1001/jamanetworkopen.2023.33251

eTable 1. *ICD-10* codes for chronic back pain and opioid use disorder from MedPAR Part A and Part B claims

eTable 2. Physical therapy and chiropractic care *CPT* codes from Part B claims

eTable 3. *ICD-10* and *CPT* codes for comorbidities from MedPAR Part A and Part B claims

eTable 4. Generic drug names for opioid prescriptions, excluding buprenorphine, in Part D claims

eTable 5. Data sources and definitions for county-level social determinants of health, geographic characteristics, and state-level practitioner availability

eTable 6. Additional characteristics of the study population by race and ethnicity

eTable 7. Characteristics of overall study population

eTable 8. Practitioner encounters after chronic low back pain diagnosis by race and ethnicity

eTable 9. All multilevel logistic regressions for the association between any physical therapy or chiropractic care and race and ethnicity

eTable 10. All multilevel logistic regressions for the association between any chiropractic care and race and ethnicity

eTable 11. All multilevel logistic regressions for the association between any physical therapy and race and ethnicity

eTable 12 All multilevel logistic regressions for the association between any physical therapy (including n=209 with evaluations only) and race and ethnicity

eTable 13. All multilevel logistic regressions for the association between any physical therapy or chiropractic care and race and ethnicity (excluding 11,887 individuals with any physical therapy or chiropractic care prior to chronic low back pain diagnosis)

This supplemental material has been provided by the authors to give readers additional information about their work.

© 2023 Bhondoekhan F et al. *JAMA Network Open*.

eTable 1: ICD-10 codes for chronic back pain and opioid use disorder from MedPAR Part A and Part B claims

Variable	Definition
Chronic radicular back pain	M4724, M4725, M4726, M4727, M4728, M5114, M5115, M5116, M5117, M5414, M5415, M5416, M5417, M5418, M5440, M5441, M5442
Chronic non-radicular back pain	M2578, M4000, M4003, M4004, M4005, M40202, M40203, M40204, M40205, M40209, M40292, M40293, M40294, M40295, M40299, M4030, M4035, M4036, M4037, M4100, M4102, M4103, M4104, M4105, M4106, M4107, M4108, M41112, M41113, M41114, M41115, M41116, M41117, M41119, M41122, M41123, M41124, M41125, M41126, M41127, M41129, M4120, M4122, M4123, M4124, M4125, M4126, M4127, M4130, M4134, M4135, M4180, M4182, M4183, M4184, M4185, M4186, M4187, M419, M4300, M4301, M4302, M4303, M4304, M4305, M4306, M4307, M4308, M4309, M4310, M4311, M4312, M4313, M4314, M4315, M4316, M4317, M4318, M4319, M4320, M4321, M4322, M4323, M4324, M4325, M4326, M4327, M4328, M438X9, M4640, M4644, M4645,M4646, M4647, M4648, M4649, M4710, M4714, M4715, M4716, M4720, M47814, M47815, M47816, M47817, M47818, M47819, M47894, M47895, M47896, M47897, M47898, M47899, M479, M4800, M4804, M4805, M4806, M48061, M48062, M4807, M4808, M4810, M4811, M4812, M4813, M4814, M4815, M4816, M4817, M4818, M4819, M4820, M4821, M4822, M4823, M4824, M4825, M4826, M4827, M4830, M4831, M4832, M4833, M4834, M4835, M4836, M4837, M4838, M489, M5104, M5105, M5106, M5124, M5125, M5126, M5127, M5134, M5135, M5136, M5137, M5146, M5147, M5184, M5185, M5186, M5187, M519, M532X7, M532X8, M533, M5380, M5384, M5385, M5386, M5387, M5388, M539, M5403, M5404, M5405, M5406, M5407, M5408, M5409, M5430, M5431, M5432, M545, M546, M5489, M549, M62830, M961, M962, M963, M965, M9922, M9923, M9924, M9925, M9926, M9927, M9928, M9929, M9932, M9933, M9934, M9935, M9936, M9937, M9938, M9939, M9942, M9943, M9944, M9945, M9946, M9947, M9948, M9949, M9952, M9953, M9954, M9955, M9956, M9957, M9958, M9959, M9962, M9963, M9964, M9965, M9966, M9967, M9968, M9969, M9972, M9973, M9974, M9975, M9976, M9977, M9978, M9979, M9983, M9984, M9902, M9904, Q762
Opioid use disorder	F1110, F1114, F1119, F1120, F1123, F1124, F1129, F1190, F1193, F1194, F1199, F11120, F11121, F11122, F11129, F11150, F11151, F11159, F11181, F11182, F11188, F11220, F11221, F11222, F11229, F11250, F11251, F11259, F11281, F11282, F11288, F11920, F11921, F11922, F11929, F11950, F11951, F11959, F11981, F11982, F11988

© 2023 Bhondoekhan F et al. *JAMA Network Open*.

eTable 2: Physical therapy and chiropractic care CPT codes from Part B claims

Current Procedural Terminology	Description
	**Evaluations**
97001	Physical therapy evaluation code used prior to January, 2017
97002	Physical therapy re-evaluation code used prior to January, 2017
97161	Physical therapy evaluation: low complexity, requiring these components:<br>A history with no personal factors and/or comorbidities that impact the plan of care; An examination of body system[s] using standardized tests and measures addressing 1–2 elements from any of the following: body structures and functions, activity limitations, and/or participation restrictions; A clinical presentation with stable and/or uncomplicated characteristics; and Clinical decision making of low complexity using standardized patient assessment instrument and/or measurable assessment of functional outcome. Typically, 20 minutes are spent face to face with the patient and/or family.
97162	Physical therapy evaluation: moderate complexity, requiring these components:<br>A history of present problem with 1–2 personal factors and/or comorbidities that impact the plan of care; An examination of body system[s] using standardized tests and measures addressing a total of 3 or more elements from any of the following: body structures and functions, activity limitations, and/or participation restrictions; An evolving clinical presentation with changing characteristics; and Clinical decision making of moderate complexity using measurable assessment of functional outcome. Typically, 30 minutes are spent face to face with the patient and/or family.
97163	Physical therapy evaluation: high complexity, requiring these components:<br>A history of present problem with 3 or more personal factors and/or comorbidities that impact the plan of care; An examination of body systems using standardized tests and measures addressing a total of 4 or more elements from any of the following: body structures and functions, activity limitations, and/or participation restrictions; A clinical presentation with unstable and unpredictable characteristics; and Clinical decision making of high complexity using standardized patient assessment instrument and/or measurable assessment of functional outcome. Typically, 45 minutes are spent face to face with the patient and/or family.
97164	Re-evaluation of physical therapy established plan of care, requiring these components:<br>An examination including a review of history and use of standardized tests and measures is required; and Revised plan of care using a standardized patient assessment instrument and/or measurable assessment of functional outcome. Typically, 20 minutes are spent face to face with the patient and/or family.
	**Modalities**
64550	Application of surface transcutaneous neurostimulator (discontinued in CPT 2019)
G0151	Home health
G0281	Electrical stimulation (unattended) to one or more areas for chronic Stage III and Stage IV pressure ulcers, arterial ulcers, diabetic ulcers, and venous stasis ulcers not demonstrating measurable signs of healing after 30 days of conventional care, as part of a therapy plan of care as maintained by CMS falls under Miscellaneous Diagnostic and Therapeutic Services
G0282	Electrical stimulation, (unattended), to one or more areas, for wound care other than described in G0281
G0283	Electrical stimulation (unattended), to one or more areas for indication(s) other than wound care, as part of a therapy plan of care
90901	Biofeedback training by any modality
97010	Hot/Cold Packs
97012	Application of a modality to one or more areas; traction, mechanical
97014	Electrical Stimulation (unattended)
97016	Vasopneumatic devices
97018	Paraffin bath
97022	Whirlpool
97024	Diathermy (e.g., microwave)
97026	Infrared
97028	Ultraviolet
97032	Application of a modality to one or more areas; electrical stimulation (manual), each 15 minutes
97033	iontophoresis, each 15 minutes
97034	contrast baths, each 15 minutes

© 2023 Bhondoekhan F et al. *JAMA Network Open*.

97035	ultrasound, each 15 minutes
97036	Hubbard tank, each 15 minutes
97039	Unlisted modality (specify type and time if constant attendance)
	**Therapeutic Procedures**
97110	Therapeutic procedure, one or more areas, each 15 minutes; therapeutic exercises to develop strength and endurance, range of motion and flexibility
97112	Neuromuscular reeducation of movement, balance, coordination, kinesthetic sense, posture, and/or proprioception for sitting and/or standing activities
97113	Aquatic therapy with therapeutic exercises
97116	Gait training (includes stair climbing)
97124	Massage, including effleurage, petrissage and/or tapotement (stroking, compression, percussion)
97139	Unlisted therapeutic procedure (specify)
97140	Manual therapy techniques (eg, mobilization/manipulation, manual lymphatic drainage, manual traction), one or more regions, each 15 minutes
97150	Group Therapy
97530	Therapeutic activities, direct (one-on-one) patient contact (use of dynamic activities to improve functional performance), each 15 minutes
97535	Self-care/home management training (eg, activities of daily living (ADL) and compensatory training, meal preparation, safety procedures, and instructions in use of assistive technology devices/adaptive equipment) direct one-on-one contact, each 15 minutes
	**Chiropractic Manipulative Treatment**
98940	Spinal, One or two Regions
98941	Spinal, Three or Four Regions
98942	Spinal, Five Regions
98943	CMT, Extraspinal, One or More Regions

© 2023 Bhondoekhan F et al. *JAMA Network Open*.

eTable 3: ICD-10 and CPT codes for comorbidities from MedPAR Part A and Part B claims

Variable	Definition
Diabetes	E08.xxx-E11.xxx, E13.xxx
Obesity	E66.01, E66.09, E66.1, E66.2, E66.3, E668, E669
Heart failure	I09.81, I11.0, I13.0, I13.2, I50.1, I50.2x, I50.3x, I50.4x, I50.81x, I50.82, I50.83, I50.84, I50.89, I50.9
Ischemic heart disease	I20, I20.0, I20.1, I20.8, I20.9, I21.01, I21.02, I21.09, I21.11, I21.19, I21.21, I21.29, I21.3, I21.4, I21.9, I21.A1, I21.A9, I22.0, I22.1, I22.2, I22.8, I22.9, I23.1, I23.2, I23.3, I23.5, I23.6, I23.7, I23.8, I24.0, I24.1, I24.8, I24.9, I25.10, I25.110, I25.111, I25.118, I25.119, I25.2, I25.3, I25.41, I25.42, I25.5, I25.6, I25.700, I25.701, I25.708, I25.709, I25.710, I25.711, I25.718, I25.719, I25.720, I25.721, I25.728, I25.729, I25.730, I25.731, I25.738, I25.739, I25.750, I25.751, I25.758, I25.759, I25.761, I25.768, I25.790, I25.798, I25.799, I25.810, I25.811, I25.812, I25.82, I25.83, I25.84, I25.89, I25.9
Depressive disorder	F32.0, F32.1, F32.2, F32.3, F32.4, F32.5, F32.89, F32.9, F33.0, F33.1, F33.2, F33.3, F33.40, F33.41, F33.42, F33.8, F33.9, F34.1
Anxiety disorder	F06.4, F40.00, F40.01, F40.02, F40.10, F40.11, F40.210, F40.218, F40.220, F40.228, F40.231, F40.232, F40.233, F40.240, F40.241, F40.242, F40.243, F40.248, F40.298, F40.8, F40.9, F41.0, F41.1, F41.3, F41.8, F41.9, F42, F42.2, F42.3, F42.4, F42.8, F42.9, F43.0, F43.10, F43.11, F43.12, F44.9
Alcohol use disorder	F10.10, F10.120, F10.121, F10.129, F10.14, F10.150, F10.151, F10.159, F10.180, F10.181, F10.188, F10.19, F10.20, F10.220, F10.221, F10.229, F10.230, F10.231, F10.232, F10.239, F10.24, F10.250, F10.251, F10.259, F10.26, F10.27, F10.280, F10.282, F10.288, F10.29, F10.920, F10.921, F10.929, F10.94, F10.950, F10.951, F10.959, F10.96, F10.97, F10.980, F10.982, F10.988, F10.99
Hypertension	H35.031, H35.032, H35.033, H35.039, I10, I11.0, I11.9, I12.0, I12.9, I13.0, I13.10, I13.11, I13.2, I15.0, I15.1, I15.2, I15.8, I15.9, I67.4
Chronic obstructive pulmonary disease	J40, J41.0, J41.1, J41.8, J42, J43.0, J43.1, J43.2, J43.8, J43.9, J44.0, J44.1, J44.9, J47.0, J47.1, J47.9
Schizophrenia or related disorder	F20-F29
Neck pain	M47811, M47891, M4723, M4722, M4721, M47813, M47893, M47892, M47812, M47012, M4711, M47014, M47011, M4712, M47022, M47029, M47019, M47016, M47013, M47021, M4713, M47015, M50220, M5022, M5023, M5020, M50221, M50222, M50223, M5021, M5002, M5000, M5001, M50022, M50023, M50021, M50020, M5003, M5082, M5091, M5012, M50922, M50821, M5080, M5011, M50123, M50822, M50820, M50921, M5010, M4643, M5093, M50120, M50823, M5083, M50121, M5090, M4641, M50920, M50122, M4642, M5092, M50923, M5013, M5081, M9930, M9971, M9921, M9970, M4803, M9931, M9940, M9960, M9950, M4802, M9951, M4801, M9961, M9941, M9920, M542, M530, M531, M5413, M5411, M5412, M436, M5400, M5402, M5401, M5382, M5481, M5381, M5383
Osteoarthritis and joint cartilage conditions	M129, M131, M138, M15, M16, M17, M18, M19, M221, M222, M223, M224, M228, M229, M23, M241
Fractures	S12, S720, S721, S722, S723, S724, S728, S729,

© 2023 Bhondoekhan F et al. *JAMA Network Open*.

	S820, S821, S822, S824, S828, S829, S823, S825, S826, S321, S322, S333, S324, S325, S326, S328, S329, S222, S223, S224, S225, S229, S420, S421, S429, S422, S423, S424, S520, S521, S522, S533, S525, S526, S529, S620, S621, S629, S140, S220, S320, S240, S340, S020, S021, S022, S023, S024, S025, S026, S028, S029, M8005, M8445, M8085, M8465, M8006, M8446, M8086, M8466, M8007, M4857, M4858, M8001, M8441, M8081, M8461, M8002, M8442, M8082, M8462, M8443, M8083, M8463, M4851, M4852, M4853, M8008, M8088, M4850, M4854, M4855, M8456, M84471, M84472, M84473, M84371, M84372, M84373, M84671, M84672, M84673
Surgery[a]	58150, 58152, 58180, 58200, 58210, 58951, 58953, 58954, 58956, 58240, 44950, 44955, 44960, 44970, 44979, 29882, 29883, 29888, 29889, 29891, 29892, 23410, 23412, 24341, 29827, 59510, 59514, 59515, 59618, 59620, 59622, 47562, 47563, 47564, 47570, 47420, 47425, 47600, 47605, 47610, 47612, 47620, 44204, 44205, 44206, 44207, 44208, 44210, 44211, 44212, 44213, 44139, 44140, 44141, 44143, 44144, 44145, 44146, 44147, 44150, 44151, 44155, 44156, 44157, 44158, 44160, 45121, 45123, 33510, 33511, 33512, 33513, 33514, 33516, 33517, 33518, 33519, 33521, 33522, 33523, , 33530, 33533, 33534, 33535, 33536, 20205, 20245, 27324, 27614, 38500, 38505, 38510, 38520, 38525, 38530, 43605, 47100, 48100, 50205, 54505,49650, 49651, 49491, 49492, 49495, 49496, 49500, 49501, 49505, 49507, 49520, 49521, 49525, 22220, 22222, 22224, 22226, 63001, 63003, 63005, 63011, 63012, 63015, 63016, 63017, 3020, 63030, 63035, 63040, 63042, 63043, 63044, 63045, 63046, 63047, 63048, 63050, 63051, 63057, 63066, 63075, 63076, 63077, 63078, 63101, 63102, 63103, 63170, 63172, 63173, 63180, 63182, 63185, 63190, 63191, 63194, 63195, 63196, 63197, 63198, 63199, 63200, 63250, 63251, 63252, 63265, 63266, 63267, 63268, 63270, 63271, 63272, 63273, 63275, 63276, 63277, 63278, 63280, 63281, 63282, 63283, 63285, 63286, 63287, 63290, 63655, 63662, 63664, 63709, 19120, 19125, 19126, 19301, 19302,19300, 19303, 19304, 60200, 60210, 60212, 60220, 60225, 60240, 60252, 60254, 60260, 60270, 60271, 31237, 31238, 31239, 31240, 31241, 31253, 31254, 31255, 31256, 31257, 31259, 31267, 31276, 31287, 31288, 31290, 31291, 31292, 31293, 31294, 31295, 31296, 31297, 31298, 22532, 22533, 22534, 22548, 22551, 22552, 22554, 22556, 22558, 22585, 22586, 22590, 2595, 22600, 22610, 22612, 22614, 22630, 22632, 22633, 22634, 22800, 22802, 22804, 22808, 22810, 22812, 22818, 22819, 22840, 22841, 22842, 22843, 22844, 22845, 22846, 22847, 22848, 22849, 22850, 22851, 22852, 22853, 22854, 22855, 22856, 22857, 22858, 22859, 22862, 22865,42820, 42821, 42825, 42826, 27090, 27091, 27125, 27130, 27132, 27134, 27137, 27138, 27437, 27438, 27440, 27441, 27442, 27443, 27445, 27446, 27447, 27486, 27487, 27488, 59400, 59409, 59410, 59610, 59612, 59614

© 2023 Bhondoekhan F et al. *JAMA Network Open*.

[a]CPT codes representing 24 procedures associated with postsurgical pain.

© 2023 Bhondoekhan F et al. *JAMA Network Open*.

eTable 4: Generic drug names for opioid prescriptions, excluding buprenorphine, in Part D claims

Generic drug name
Acetaminophen-Codeine
Acetaminophen-Hydrocodone
Acetaminophen-Oxycodone
Acetaminophen-Tramadol
Acetaminophen/Butalbital/Caffeine/Codeine
Acetaminophen/Caffeine/Dihydrocodeine
Aspirin-Oxycodone
Aspirin/Butalbital/Caffeine/Codeine
Belladonna-Opium
Butorphanol Codeine
Fentanyl
Hydrocodone
Hydrocodone-Ibuprofen
Hydromorphone
Ibuprofen-Oxycodone
Levorphanol
Meperidine
Methadone[a]
Morphine
Morphine-Naltrexone
Naloxone-Pentazocine
Oxycodone
Oxymorphone
Tapentadol
Tramadol

[a]Medicare did not cover methadone for OUD under Part D until 2020. Therefore, methadone identified in Part D during our study period (2016-2018) is expected to be for pain management.

© 2023 Bhondoekhan F et al. *JAMA Network Open*.

eTable 5: Data sources and definitions for county-level social determinants of health, geographic characteristics, and state-level practitioner availability

Variable	Source	Definition
% with less than 12 years of education	2014-2018 ACS 5-year county-level summary file: B15003	% population 25 years or more with less than 12 years of education = (population with less than high school diploma or 12 years of education) / (total population)
% single-parent household	2014-2018 ACS 5-year county-level summary files: B11003	% single-parent households with dependents < 18 years = (total single-parent households (male and female) with dependents <18 years) / (total population)
% living in the rented housing units	2014-2018 ACS 5-year county-level summary file: B25003	% population living in renter-occupied housing units = (renter occupied housing units) / (owner-occupied housing units + renter occupied housing units)
% living in the overcrowded housing units	2014-2018 ACS 5-year county-level summary files: B25014	% population living in crowded housing units = (tenure by occupants per room – a population with ≥ 1.01 occupants per room in owner-occupied housing units and Renter occupied housing units) / (total population)
% of households without a car	2014-2018 ACS 5-year county-level summary files: B25044	% population with no car = (population with no vehicle available) / (total population)
% high needs population	2014-2018 ACS 5-year county-level summary file: B01001	% high needs population = (population under 5 years of age + women between the ages of 15-44 years + everyone 65 years and over) / (total population)
% non-employed population (civilian)	2014-2018 ACS 5-year county-level summary files: B23001	% Non-employed = (not in labor force + unemployed) / (civilian + not in the labor force for the population 16-64 years)
Urbanicity	2020-2021 AHRF county-level data	F0002013 = "Rural-Urban Continuum Code 2013" (metropolitan, urban, rural)
US region	2020-2021 AHRF county-level data	F04448 = "Census Region Name"
Number of chiropractors per 100,000 population	2020-2021 AHRF state and national-level data	SF14201-19
Number of physical therapists or assistants or aides per 100,000 population	2020-2021 AHRF state and national-level data	SF26201-19, SF27201-19

© 2023 Bhondoekhan F et al. *JAMA Network Open*.

eTable 6. Additional characteristics of the study population by race and ethnicity[a]

	American Indian or Alaska Native	Asian or Pacific Islander	Black or African American	Hispanic	non-Hispanic White	Unknown or other[b]
N	745	444	9822	4124	53377	850
**Medicare eligibility**						
Current reason for eligibility						
Old Age and Survivors Insurance (OASI), No. (%)	217 (29.1)	226 (50.9)	2685 (27.3)	1310 (31.8)	21869 (41.0)	393 (46.2)
Disability Insurance Benefits (DIB), No. (%)	518 (69.5)	215 (48.4)	6982 (71.1)	2766 (67.1)	31363 (58.8)	452 (53.2)
Dual eligibility	224 (30.1)	106 (23.9)	2737 (27.9)	1156 (28.0)	10268 (19.2)	155 (18.2)
**County-level social determinants of health**						
N	298	203	1154	773	2739	479
% high needs population, median (IQR)	40.8 (39.7–42.0)	40.5 (39.6–41.7)	41.0 (39.8–42.1)	40.8 (39.7–42.0)	41.1 (39.9–42.3)	40.9 (39.7–41.9)
% non-employed population, median (IQR)	33.3 (28.8–39.0)	30.3 (26.9–34.3)	35.2 (30.2–40.5)	32.6 (28.5–37.9)	33.7 (28.3–40.0)	31.0 (27.5–36.3)
% living in the rented housing units, median (IQR)	31.3 (26.1–37.1)	35.5 (28.8–40.8)	30.6 (25.9–36.6)	31.3 (26.5–37.1)	27.6 (23.5–32.7)	31.0 (26.1–37.3)
% with less than 12 years of education, median (IQR)	8.1 (5.8–10.2)	7.6 (5.8–10.0)	9.6 (6.8–12.9)	8.2 (6.4–11.3)	8.8 (6.4–12.3)	7.5 (5.8–9.7)
% single-parent household, median (IQR)	3.4 (2.9–3.8)	3.3 (2.8–3.8)	3.5 (3.0–4.1)	3.4 (2.9–4.0)	3.3 (2.7–3.8)	3.3 (2.8–3.9)
% of households without a car, median (IQR)	2.3 (1.8–2.8)	2.4 (1.7–3.3)	2.5 (1.9–3.4)	2.3 (1.7–3.0)	2.3 (1.7–3.0)	2.4 (1.8–3.2)
% living in overcrowded housing units, median (IQR)	1.0 (0.7–1.4)	0.9 (0.6–1.5)	0.8 (0.5–1.1)	0.9 (0.6–1.3)	0.7 (0.5–1.1)	0.7 (0.5–1.1)
**State-level practitioner availability (per 100,000 population)**						
N	42	44	49	50	51	50
Number of physical therapists or assistants or aides, median (IQR)	112.7 (103.8–131.4)	113.2 (103.3–131.9)	113.2 (104.7– 132.4)	115.4 (104.7–134.1)	117.5 (104.7–136.4)	115.4 (104.7–134.1)
Number of chiropractors, median (IQR)	18.7 (13.7–28.8)	18.4 (14.0–25.9)	18.6 (14.4–26.8)	18.7 (14.4–26.8)	18.6 (14.4–26.8)	18.4 (14.4–25.9)

[a]All characteristics assessed in the year of chronic low back pain diagnosis.
[b]Other refers to non-Hispanic other races and any missing values are coded as unknown.

© 2023 Bhondoekhan F et al. *JAMA Network Open*.

eTable 7. Characteristics of overall study population

	Beneficiaries, No. (%)
N	69362
**Demographic**	
Age	
Median (IQR)	60.0 (51.5-68.7)
≥65 years	26189 (37.8)
**Sex**	
Female	42042 (60.6)
Male	27320 (39.4)
**Comorbidities**[a]	
Cardiovascular condition[b]	44563 (64.2)
Mental health diagnosis[c]	35316 (50.9)
Osteoarthritis and joint cartilage conditions	25245 (36.4)
Neck pain	23744 (34.2)
Fractures	3858 (5.6)
Diabetes	19942 (28.8)
Chronic obstructive pulmonary disease	16827 (24.3)
Obesity	12985 (18.7)
Alcohol use disorder	2772 (4.0)
Hospitalization	11712 (16.9)
Surgery[d]	3464 (5.0)
Gagne comorbidity score	
≤0	27788 (40.1)
1	15690 (22.6)
2-3	15693 (22.6)
≥4	10191 (14.7)
**Medication**	
Opioid prescription[a]	61048 (88.0)
MOUD[e]	3845 (5.5)
**Geography**[e]	
Urbanicity	
Metropolitan	51963 (74.9)
Urban	15237 (22.0)
Rural	2162 (3.1)
US Region	
Midwest	12786 (18.4)
Northeast	10402 (15.0
South	34168 (49.3)
West	12006 (17.3)

Abbreviations: MOUD, medication for opioid use disorder.
[a]Assessed 6 months prior to the date of chronic low back pain diagnosis.
[b]Defined as a diagnosis of heart failure, ischemic heart disease, or hypertension.
[c]Defined as a diagnosis of depressive disorder, anxiety disorder, or schizophrenia or related disorder.
[d]Refers to 24 procedures associated with postsurgical pain.
[e]Assessed in the year of chronic low back pain diagnosis.

© 2023 Bhondoekhan F et al. *JAMA Network Open*.

15 **eTable 8: Practitioner encounters after chronic low back pain diagnosis by race and ethnicity**

	Beneficiaries, No. (%)					
	American Indian or Alaska Native	Asian or Pacific Islander	Black or African American	Hispanic	Non-Hispanic White	Unknown or other[a]
N	745	444	9822	4124	53377	850
Chiropractor	70 (9.4)	54 (12.2)	654 (6.7)	323 (7.8)	6753 (12.7)	107 (12.6)
Pain medicine specialist	289 (38.8)	172 (38.7)	4084 (41.6)	1678 (40.7)	20623 (38.6)	331 (38.9)
Physical rehabilitation practitioner	104 (14.0)	76 (17.1)	1374 (14.0)	613 (14.9)	7537 (14.1)	119 (14.0)
Physical therapist	57 (7.7)	69 (15.5)	844 (8.6)	431 (10.5)	5490 (10.3)	102 (12.0)
Addiction specialist	23 (3.1)	9 (2.0)	137 (1.4)	52 (1.3)	1039 (1.9)	19 (2.2)
Other practitioner specialty	722 (96.9)	432 (97.3)	9602 (97.8)	4046 (98.1)	51627 (96.7)	812 (95.5)

16 [a]Other refers to non-Hispanic other races and any missing values are coded as unknown.

© 2023 Bhondoekhan F et al. *JAMA Network Open*.

eTable 9: All multilevel logistic regressions for the association between any physical therapy or chiropractic care and race and ethnicity[a]

	Model 1	Model 2	Model 3	Model 4	Model 5	Model 6	Model 7
	OR (95% CI)[b]	aOR (95% CI)	aOR (95% CI)	aOR (95% CI)	aOR (95% CI)	aOR (95% CI)	aOR (95% CI)
American Indian or Alaska Native	0.64** (0.48–0.85)	0.74 (0.55–1.00)	0.75 (0.55–1.01)	0.76 (0.56–1.03)	0.73* (0.54–0.99)	0.74* (0.54–1.00)	0.74* (0.54–0.99)
Asian or Pacific Islander	1.06 (0.80–1.41)	1.03 (0.75–1.41)	0.98 (0.72–1.35)	0.96 (0.70–1.31)	0.96 (0.70–1.31)	0.96 (0.70–1.31)	0.96 (0.70–1.31)
Black or African American	0.66*** (0.61–0.72)	0.75*** (0.68–0.82)	0.76*** (0.69–0.84)	0.77*** (0.70–0.85)	0.78*** (0.71–0.86)	0.79*** (0.71–0.87)	0.79*** (0.71–0.87)
Hispanic	0.69*** (0.61–0.77)	0.81*** (0.71–0.92)	0.81*** (0.71–0.92)	0.83** (0.73–0.94)	0.84** (0.74–0.95)	0.84** (0.74–0.96)	0.84** (0.74–0.96)
non-Hispanic White	Ref	Ref	Ref	Ref	Ref	Ref	Ref
Unknown or other[c]	1.17 (0.96–1.43)	1.15 (0.91–1.43)	1.10 (0.87–1.38)	1.08 (0.86–1.36)	1.09 (0.87–1.37)	1.08 (0.86–1.36)	1.08 (0.86–1.36)

[a]Model 1: unadjusted; Model 2: Model 1 + demographic characteristics + reason for Medicare eligibility + dual eligibility + any PT or chiropractic care before chronic low back pain diagnosis; Model 3: Model 2 + comorbidities + medication; Model 4: Model 3 + social determinants of health; Model 5: Model 4 + state-level practitioner availability; Model 6: Model 5 + urbanicity + US region; Model 7: Model 6 + addiction specialist encounters during follow-up. All models accounted for clustering by county and state.

[b]Odds ratio with 95% confidence intervals in parentheses.

[c]Other refers to non-Hispanic other races and any missing values are coded as unknown.

* $p<0.05$, ** $p<0.01$, *** $p<0.001$.

© 2023 Bhondoekhan F et al. *JAMA Network Open*.

eTable 10: All multilevel logistic regressions for the association between any chiropractic care and race and ethnicity[a]

	Model 1	Model 2	Model 3	Model 4	Model 5	Model 6	Model 7
	OR (95% CI)[b]	aOR (95% CI)	aOR (95% CI)	aOR (95% CI)	aOR (95% CI)	aOR (95% CI)	aOR (95% CI)
American Indian or Alaska Native	0.54** (0.36–0.81)	0.66 (0.43–1.01)	0.68 (0.44–1.05)	0.70 (0.46–1.08)	0.68 (0.44–1.05)	0.69 (0.45–1.06)	0.69 (0.45–1.06)
Asian or Pacific Islander	0.71 (0.43–1.16)	0.73 (0.43–1.23)	0.71 (0.42–1.20)	0.77 (0.46–1.30)	0.77 (0.45–1.30)	0.77 (0.45–1.29)	0.77 (0.45–1.29)
Black or African American	0.36*** (0.30–0.42)	0.39*** (0.33–0.47)	0.42*** (0.36–0.50)	0.45*** (0.38–0.54)	0.45*** (0.38–0.54)	0.46*** (0.39–0.55)	0.46*** (0.39–0.55)
Hispanic	0.42*** (0.34–0.52)	0.49*** (0.39–0.61)	0.49*** (0.40–0.62)	0.53*** (0.42–0.66)	0.53*** (0.42–0.66)	0.54*** (0.43–0.68)	0.54*** (0.43–0.67)
non-Hispanic White	Ref	Ref	Ref	Ref	Ref	Ref	Ref
Unknown or other[c]	1.05 (0.79–1.41)	0.93 (0.68–1.28)	0.88 (0.64–1.21)	0.90 (0.66–1.24)	0.91 (0.66–1.25)	0.89 (0.65–1.23)	0.90 (0.65–1.23)

[a]Model 1: unadjusted; Model 2: Model 1 + demographic characteristics + reason for Medicare eligibility + dual eligibility + any PT or chiropractic care before chronic low back pain diagnosis; Model 3: Model 2 + comorbidities + medication; Model 4: Model 3 + social determinants of health; Model 5: Model 4 + state-level practitioner availability; Model 6: Model 5 + urbanicity + US region; Model 7: Model 6 + addiction specialist encounters during follow-up. All models accounted for clustering by county and state.
[b]Odds ratio with 95% confidence intervals in parentheses.
[c]Other refers to non-Hispanic other races and any missing values are coded as unknown.
** p<0.01, *** p<0.001.

© 2023 Bhondoekhan F et al. *JAMA Network Open*.

eTable 11: All multilevel logistic regressions for the association between any physical therapy and race and ethnicity[a]

	Model 1	Model 2	Model 3	Model 4	Model 5	Model 6	Model 7
	OR (95% CI)[b]	aOR (95% CI)	aOR (95% CI)	aOR (95% CI)	aOR (95% CI)	aOR (95% CI)	aOR (95% CI)
American Indian or Alaska Native	0.73 (0.51–1.05)	0.82 (0.56–1.20)	0.82 (0.56–1.19)	0.80 (0.55–1.17)	0.80 (0.55–1.16)	0.81 (0.55–1.17)	0.81 (0.55–1.17)
Asian or Pacific Islander	1.26 (0.92–1.73)	1.22 (0.87–1.71)	1.17 (0.83–1.64)	1.08 (0.76–1.52)	1.08 (0.76–1.52)	1.07 (0.76–1.51)	1.07 (0.76–1.51)
Black or African American	0.89* (0.80–0.98)	1.05 (0.95–1.17)	1.03 (0.93–1.15)	1.02 (0.92–1.13)	1.02 (0.92–1.14)	1.02 (0.92–1.14)	1.02 (0.92–1.14)
Hispanic	0.85* (0.74–0.97)	1.03 (0.90–1.19)	1.01 (0.88–1.17)	1.01 (0.87–1.17)	1.01 (0.87–1.17)	0.99 (0.86–1.15)	1.00 (0.86–1.15)
non-Hispanic White	Ref	Ref	Ref	Ref	Ref	Ref	Ref
Unknown or other[c]	1.24 (0.97–1.58)	1.26 (0.97–1.63)	1.23 (0.95–1.60)	1.19 (0.92–1.55)	1.19 (0.92–1.55)	1.20 (0.92–1.55)	1.20 (0.92–1.55)

[a]Model 1: unadjusted; Model 2: Model 1 + demographic characteristics + reason for Medicare eligibility + dual eligibility + any PT or chiropractic care before chronic low back pain diagnosis; Model 3: Model 2 + comorbidities + medication; Model 4: Model 3 + social determinants of health; Model 5: Model 4 + state-level practitioner availability; Model 6: Model 5 + urbanicity + US region; Model 7: Model 6 + addiction specialist encounters during follow-up. All models accounted for clustering by county and state.
[b]Odds ratio with 95% confidence intervals in parentheses.
[c]Other refers to non-Hispanic other races and any missing values are coded as unknown.
* $p<0.05$.

© 2023 Bhondoekhan F et al. *JAMA Network Open*.

eTable 12: All multilevel logistic regression for the association between any physical therapy (including n=209 with evaluations only) and race and ethnicity[a]

	Model 1	Model 2	Model 3	Model 4	Model 5	Model 6	Model 7
	OR (95% CI)[b]	aOR (95% CI)	aOR (95% CI)	aOR (95% CI)	aOR (95% CI)	aOR (95% CI)	aOR (95% CI)
American Indian or Alaska Native	0.76 (0.54–1.08)	0.83 (0.58–1.19)	0.82 (0.57–1.18)	0.81 (0.57–1.17)	0.81 (0.56–1.16)	0.82 (0.57–1.17)	0.82 (0.57–1.17)
Asian or Pacific Islander	1.26 (0.92–1.71)	1.22 (0.87–1.70)	1.17 (0.84–1.64)	1.08 (0.77–1.51)	1.08 (0.77–1.51)	1.08 (0.77–1.51)	1.08 (0.77–1.50)
Black or African American	0.89* (0.81–0.98)	1.05 (0.95–1.16)	1.04 (0.94–1.15)	1.02 (0.92–1.14)	1.03 (0.92–1.14)	1.03 (0.92–1.14)	1.03 [0.92–1.14)
Hispanic	0.87* (0.76–0.99)	1.04 (0.91–1.20)	1.03 (0.89–1.18)	1.02 (0.89–1.18)	1.02 (0.89–1.18)	1.01 (0.88–1.16)	1.01 (0.88–1.16)
non-Hispanic White	Ref	Ref	Ref	Ref	Ref	Ref	Ref
Unknown or other[c]	1.24 (0.98–1.57)	1.26 (0.97–1.62)	1.23 (0.95–1.58)	1.19 (0.92–1.53)	1.19 (0.92–1.53)	1.19 (0.92–1.54)	1.19 (0.92–1.54)

[a]Model 1: unadjusted; Model 2: Model 1 + demographic characteristics + reason for Medicare eligibility + dual eligibility + any PT or chiropractic care before chronic low back pain diagnosis; Model 3: Model 2 + comorbidities + medication; Model 4: Model 3 + social determinants of health; Model 5: Model 4 + state-level practitioner availability; Model 6: Model 5 + urbanicity + US region; Model 7: Model 6 + addiction specialist encounters during follow-up. All models accounted for clustering by county and state.
[b]Odds ratio with 95% confidence intervals in parentheses.
[c]Other refers to non-Hispanic other races and any missing values are coded as unknown.
* p<0.05.

© 2023 Bhondoekhan F et al. *JAMA Network Open*.

eTable 13: All multilevel logistic regression for the association between any physical therapy or chiropractic care and race and ethnicity (excluding 11,887 individuals with any physical therapy or chiropractic care prior to chronic low back pain diagnosis)[a]

	Model 1	Model 2	Model 3	Model 4	Model 5	Model 6	Model 7
	OR (95% CI)[b]	aOR (95% CI)	aOR (95% CI)	aOR (95% CI)	aOR (95% CI)	aOR (95% CI)	aOR (95% CI)
American Indian or Alaska Native	0.80 (0.52–1.22)	0.85 (0.56–1.29)	0.83 (0.55–1.27)	0.84 (0.55–1.28)	0.80 (0.52–1.22)	0.82 (0.53–1.25)	0.82 (0.53–1.25)
Asian or Pacific Islander	1.26 (0.81–1.95)	1.26 (0.81–1.96)	1.21 (0.78–1.89)	1.16 (0.74–1.81)	1.16 (0.74–1.81)	1.16 (0.74–1.80)	1.16 (0.74–1.80)
Black or African American	0.77*** (0.67–0.88)	0.82** (0.71–0.93)	0.80** (0.70–0.92)	0.82** (0.71–0.94)	0.83* (0.73–0.96)	0.84* (0.73–0.96)	0.84* (0.73–0.96)
Hispanic	0.81* (0.67–0.97)	0.87 (0.72–1.04)	0.85 (0.71–1.02)	0.89 (0.74–1.07)	0.89 (0.74–1.07)	0.88 (0.73–1.06)	0.88 (0.73–1.06)
non-Hispanic White	Ref	Ref	Ref	Ref	Ref	Ref	Ref
Unknown or other[c]	1.13 (0.81–1.59)	1.10 (0.79–1.55)	1.05 (0.74–1.47)	1.03 (0.73–1.45)	1.03 (0.73–1.45)	1.03 (0.73–1.45)	1.03 (0.73–1.45)

[a]Model 1: unadjusted; Model 2: Model 1 + demographic characteristics + reason for Medicare eligibility + dual eligibility; Model 3: Model 2 + comorbidities + medication; Model 4: Model 3 + social determinants of health; Model 5: Model 4 + state-level practitioner availability; Model 6: Model 5 + urbanicity + US region; Model 7: Model 6 + addiction specialist encounters during follow-up. All models accounted for clustering by county and state.
[b]Odds ratio with 95% confidence intervals in parentheses.
[c]Other refers to non-Hispanic other races and any missing values are coded as unknown.
* p<0.05, ** p<0.01, *** p<0.001.

© 2023 Bhondoekhan F et al. *JAMA Network Open*.
