# Supplementary material for: Racial and Ethnic Differences in Receipt of Nonpharmacologic Care for Chronic Low Back Pain Among Medicare Beneficiaries With OUD
Source: JAMA Netw Open. 2023 Sep 12;6(9):e2333251. doi: 10.1001/jamanetworkopen.2023.33251 (PMC10498328; doi:10.1001/jamanetworkopen.2023.33251)
Supplement: Supplement 2. — Data Sharing Statement [file jamanetwopen-e2333251-s002.pdf]

# Data Sharing Statement

Bhondoekhan. Racial and Ethnic Differences in Receipt of Nonpharmacologic Care for Chronic Low Back Pain Among Medicare Beneficiaries With OUD. *JAMA Netw Open*. Published September 12, 2023. doi:10.1001/jamanetworkopen.2023.33251

**Data**
**Data available:** No. The data use agreement does not allow data sharing.
